# The effect of pulsatile motion and cardiac-gating on reconstruction and diffusion tensor properties of the corticospinal tract

**DOI:** 10.1038/s41598-018-29525-0

**Published:** 2018-07-25

**Authors:** Miriam H. A. Bopp, Jia Yang, Christopher Nimsky, Barbara Carl

**Affiliations:** Philipps University Marburg, Department of Neurosurgery, Baldingerstrasse, Marburg, 35043 Germany

## Abstract

Pulsatile motion occurs in the cardiac systolic period and leads to significantly larger displacement of water molecules as it is observed during diffusion weighted image acquisition. Obvious pulsatile motion arises in the brain stem and basal ganglia and might affect the corticospinal tract. So far there is no consensus on the real effect of pulsatile motion on diffusion properties, diffusion tensor parameters and fiber tractography and on the role of cardiac-gating to overcome these effects. The present study aimed at detecting effects of pulsatile motion on imaging properties and reconstruction of the corticospinal tract. Non-gated and cardiac-gated data of 22 healthy subjects was acquired using clinical standard protocols and analysed with regard to effects on signal intensities, diffusion tensor properties and tractography results concerning the corticospinal tract. Analyses resulted in obvious effects of pulsatile motion on signal intensities, especially alterations in diffusion tensor properties, compensated by the application of cardiac-gating, whereas no effect on fiber tract volume was seen. Therefore, pulsatile motion and cardiac-gating should be kept in mind as critical aspects when analysing and interpreting diffusion tensor properties within the human brain, but are of minor interest when considering fiber tractography of the corticospinal tract.

## Introduction

Diffusion Tensor Imaging (DTI) based fiber tractography has become a routine tool to estimate the course, location and spatial extent of major white matter tracts, such as the corticospinal tract (CST), in neurosurgical applications. DTI applications have been shown to support the concept of maximized tumour volume reduction whilst preserving neurological functions thereby contributing to low postoperative morbidity^1–4^. Even though the integration of DTI based fiber tractography in multimodal neuronavigation shows significant benefits, especially the spatial extent of reconstructed white matter tracts is often underestimated and leads to higher data uncertainty^5,6^. In order to further reduce uncertainty, several aspects of the image processing pipeline can be addressed. These aspects range from data acquisition, being prone to various artefacts e.g. due to pulsatile motion, and the calculation of the diffusion profile, using a simple and limited tensor model, to the choice of suitable fiber tracking algorithms and visualization techniques.

Brownian motion of water molecules can be described as a kind of micro-motion with displacements of about 10 µm/50 ms^7^. During MRI data acquisition, additional types of motion arise such as voluntary head bulk motion and physiological pulsatile motion. Head bulk motion can be reduced by optimized positioning approaches, e.g. using soft foam pads to fixate the head and post-processing tools for further reduction of head bulk motion. Pulsatile motion occurs in the cardiac systolic period and is contributed by artery pulse, venous expansion, cerebral spinal fluid (CSF) flow and capillary expansion^8^. The cerebral artery pulse is the primary power for pulsatile motion^9^. Reports show displacements of about 100 µm–184 µm/50 ms according to pulsatile motion, much larger than the observed motion of water molecules during diffusion weighted image (DWI) data acquisition^7,10^. Highest velocities occur in the brain stem (1.5 mm/s) in anterio-caudal direction and within the basal ganglia (1.0 mm/s) in medial-caudal and posterior-caudal direction^9^. As DWI is sensitive to motion, in particular Brownian motion, it is also affected by brain pulsatile motion, superimposing Brownian motion. In order to reduce the effects of pulsatile motion occurring during the systolic period, cardiac-gated or peripheral pulse-gated data acquisition has been suggested with acquisition of MRI data only in the diastolic period^11,12^.

Up to now, there is no consensus on the real effect of brain pulsatile motion on the diffusion signal, diffusion tensor parameters and fiber tractography and on the role of cardiac-gating to overcome these effects. While some previous studies show that prominent signal artefacts in non-gated DWI data can be eliminated by cardiac-gating^7,13^, other studies report less prominent effects with an artefact occurrence ratio of around 6% to 20%^10,12^ or even could not detect obvious artefacts at all within the non-gated DWI data^14^. Besides obvious artefacts, some studies also showed less effects on diffusion tensor parameters such as fractional anisotropy (FA) and mean diffusivity (MD) in cardiac-gated DWI data, thereby considering cardiac-gating as a helpful tool^7,12,13,15–17^. Apart from the effects of pulsatile motion on scalar diffusion tensor parameters (e.g. FA), additional effects on the principal eigenvector’s direction, relevant for fiber tractography, were reported^18,19^. Up to now, only a single study focused on the effects of pulsatile motion on white matter tractography with regard to the fornix and the corpus callosum^18^. Although there is strong evidence for the advantages of cardiac-gated DWI over non-gated DWI data acquisition, it is not usually considered in the clinical routine due to variable acquisition times depending on the heart rate, frequently becoming considerably longer^10^.

As the most obvious pulsatile motion is reported to occur within the brain stem and basal ganglia, the corticospinal tract is likely to be affected by pulsatile motion. As this major white matter tract is of great importance in neurosurgical interventions, the effects of pulsatile motion and cardiac-gated DWI data acquisition on diffusion tensor parameters and fiber tractography are analysed in this study.

## Methods

### Volunteers

In total, 22 healthy subjects (mean age: 27.45 ± 6.10 years, male/female ratio: 9/13) were included in a prospectively designed study on effects of cardiac-gated DWI on fiber tractography reliability of the corticospinal tract. MRI data of all subjects was acquired during a single session. All subjects were right-handed and completed the equivalent of a high-school degree. No one of them had any confirmed organic brain diseases, any severe medical and/or neurological condition of lifetime substance dependence. General exclusion criteria were any common contraindications for MRI data acquisition such as pace maker and/or metallic implants.

Written informed consent was provided by all subjects after complete description of the study procedures (experimental setting, mechanism and risks of MRI data acquisition). The study protocol was approved by the local ethics committee at the Philipps University Marburg in accordance to the declaration of Helsinki (reference number 162/12). MRI methods were carried out in accordance with the approved guidelines.

### MRI Data Acquisition

All MRI data sets were acquired at a 3 T MRI System (Tim Trio, Siemens, Erlangen, Germany) equipped with a 12-channel head matrix Rx-coil at Philipps University Marburg. Data acquisition included a T1-weighted magnetization prepared rapid gradient echo (MPRAGE) sequence and ten subsequent sets of DWI, five non-gated and five cardiac-gated acquisition schemes, using a single-shot echo planar imaging sequence with parameters as follows:

T1-MPRAGE: repetition time (TR) 1900 ms, echo time (TE) 2.26 ms, inversion time (TI) 900 ms, Field of View (FoV) 256 mm, matrix 256 × 256, slice thickness (ST) 1 mm, distance factor 50%, flip angle 9°, 176 slices, parallel imaging (GRAPPA) with factor 2

DWI: TR 7800 ms (non-gated)/around 700 ms (cardiac-gated, according to individual heart rate), TE 90 ms, FoV 256 mm, matrix 128 × 128, ST 2 mm distance factor 0%, 40 slices, GRAPPA with factor 2, 30 diffusion encoding gradients, high-b-value 1000 s/mm², axial slices, phase encoding direction anterior» posterior, resulting voxel size: 2 × 2 × 2 mm³

Data acquisition times for non-gated DWI were about 6 minutes per excitation whereas acquisition times for cardiac-gated DWI data took slightly longer with about 9.92 ± 2.02 minutes per excitation.

The measurement volume of DWI was aligned in parallel to the connecting line of anterior and posterior commissure (sagittal section) and in parallel to the midsagittal plane, covering the entire cerebrum. Each data set was visually inspected and subjects would have been excluded if at least one volume showed severe artefacts or alterations. However, in this study, no severe artefacts or alterations were seen.

During MRI data acquisition, subjects were positioned with head first and in a supine position in the MRI scanner in a dimmed environment using no light inside the scanner and 100% white light within the scanner environment. Subjects’ arms were positioned comfortably beside the body. The subject’s head was oriented in prolongation of the body line and fixated with soft foam rubber pads to minimize head movements and standardize head position. Furthermore, the nasal bone of the subject was positioned at the isocenter of the magnetic field.

### Data analysis

To account for variability or dissimilarity across the repeated measurements the DWI data sets were not averaged for further processing. At the different levels of analysis averaging of the current parameters across the repeated measurements was applied.

### Motion artefacts and diffusion weighted signal intensities

To evaluate motion artefacts due to pulsatile motion all DWI data sets were first corrected for head bulk motion and eddy current artefacts using “eddy”, a tool for correcting eddy currents and subject motion in diffusion data^20^ implemented in FSL (FMRIB Software Library, Oxford, United Kingdom)^21–23^. In addition, the DWI data sets were co-registered onto the individual T1-weighted image set using FLIRT (FMRIB’s Linear Image Registration Tool, Oxford, UK)^24–26^. To estimate pulsatile motion artefacts, the DWI data sets were observed qualitatively. According to previous literature^9,10^ most obvious artefacts are expected when the diffusion encoding gradient direction equals the direction of pulsatile motion. In this way previous reports demonstrated pronounced artefacts in the brain stem and the cerebellum in case of diffusion encoding gradient direction along the z-axis^7,12,27^. This approximation only holds if the brain stem is positioned in parallel to the z-axis. In order to analyse pulsatile motion artefacts in this area for specific diffusion encoding gradient directions, the subspace angles of the diffusion encoding gradient directions to the z-axis were calculated (the cosine of the subspace angle equals the dot product of diffusion encoding gradient direction and z-axis divided by the product of their vector magnitudes). For further analysis we therefore chose the diffusion encoding gradient direction with smallest subspace angle (18.77° ± 5.42°, diffusion encoding gradient #24, named volume 24 in the following paragraphs) closest to the z-axis and largest subspace angle (95.52° ± 2.35°, diffusion encoding gradient #17, named volume 17 in the following paragraphs).

The five non-gated and five cardiac-gated as well as the averaged non-gated and cardiac-gated DWI volumes of both selected diffusion encoding gradients were visually inspected for obvious pulsatile motion artefacts. In correspondence to previous studies obvious signal loss or signal attenuation was considered as obvious pulsatile motion artefact^7,28^.

To further evaluate the consistency and variability of the DWI signal intensity, standard deviation (SD) maps were calculated for each volunteer representing the SD of DWI signal intensity across the non-gated and across the cardiac-gated image sets. For group level analysis, all T1-weighted images were co-registered to the MNI152 (Montreal Neurological Institute) standard space using FLIRT and all SD maps were transformed accordingly. Group level SD maps were calculated for the non-gated and cardiac-gated data sets as well as a difference SD map between both groups.

### Diffusion tensor properties and parameters

In order to estimate and analyse diffusion tensor parameters of the co-registered DWI data sets (single subject level) such as FA, MD, the three eigenvalues L1, L2, L3 and the principal eigenvector V1, the corrected (motion and eddy currents) DWI data sets were processed using *dtifit* within the FSL Diffusion Toolbox (FDT). In addition, all created image sets were normalized to the MNI152 (Montreal Neurological Institute) template using FLIRT. To mask the whole CST within the template space, the JHU white matter tractography atlas^29–31^ was utilized. Average FA and MD maps were generated for the non-gated and cardiac-gated data sets. Mean FA and MD was calculated within the masked tracts for each volunteer and non-gated and cardiac-gated DWI data sets. To further acquire local changes within the tract, the CST was also subdivided into 5 mm slabs ranging from pons to the motor cortex. Mean FA and MD is then calculated for every slab.

### Tract based spatial statistics (TBSS) analysis

To allow for analyses of general alterations on group level, a TBSS analysis^23,32–34^ was set up. For comparison between both groups (non-gated vs. cardiac-gated) for each volunteer and each diffusion parameter an average diffusion parameter map (FA, MD, L1, L2, L3) was calculated out of the corresponding five maps per parameter. First, all FA data was normalized using the standard MNI space with non-linear registration^34^. All transformed data sets were subsequently resampled, resulting in a spatial resolution of 1 × 1 × 1 mm³. Second, a mean FA-image was created and thinned to generate a mean FA skeleton. Finally, all aligned FA data sets were projected onto the mean FA skeleton using a non-maximum suppression threshold of FA ≥0.20. The resulting data was then used for voxel-wise analyses.

Voxel-wise statistical analyses of differences between non-gated and cardiac-gated image sets were performed on the whole-brain mean FA skeleton to identify significantly affected white matter tracts using a general linear model (GLM), set up in the FSL Randomize Tool^35,36^. The contrasts (non-gated > cardiac-gated, non-gated < cardiac-gated) were analysed according to permutation-based non-parametric inference with 500 random permutations using threshold-free cluster enhancement (TFCE)^37^ to correct for multiple testing. Significance level was set to p < 0.05. Moreover, to analyse effects on other diffusion tensor parameters such as MD and the three eigenvalues, all corresponding maps were transformed accordingly, projected on the FA skeleton and processed comparable to the FA data.

### Analysis of the principal eigenvector

A TBSS analysis was set up to analyse the effect of pulsatile motion on the principal eigenvector V1. Therefore, the angle between the principal eigenvector and the z-axis was calculated (see calculation of subspace angles) for each voxel and the resulting maps were used for TBSS analysis as described before (see TBSS analysis of MD maps). As the CST roughly follows the z-axis, only the angle to the z-axis was chosen.

In addition the created angle maps of each group (non-gated vs. cardiac-gated) were evaluated within 5 mm slabs along the corticospinal tract to further analyse the impact of pulsatile motion on the eigenvector’s direction.

### Fiber tractography of the CST

For fiber tractography of the CST, all original DWI data sets including the corresponding T1-weighted data set were transferred to the neuronavigation software platform iPlan (Brainlab, Munich, Germany). Within this standard neuronavigation software, motion and eddy current correction was performed on the data sets and all DWI data sets were affine registered to the T1-weighted image. To outline the CST two include regions of interest were defined on the T1-weighted image, one in the motor cortex and one in the cerebral peduncle, and were used for fiber tractography in all DWI data sets. After fiber tractography, several exclude regions were defined to delete reconstructed parts not belonging to the CST. These exclude regions were also defined on the T1-weighted data set and used for all DWI data sets. For all reconstructed fiber tracts corresponding objects were generated and exported for further analysis.

### Tract variability using Jaccard Distance

The Jaccard coefficient (JC), also known as Intersection over Union measure, is a normalized measure of overlap typically used to compare two sample sets and is defined as the size of the intersection divided by the size of the union of both samples^38^. As in this case we focus on the tract variability the complementary measure, the Jaccard distance (JD) is used. The JD defined as JD = 1 − JC is a measure for dissimilarity between finite data sets and is in this case used in a more generalized form (five samples) to analyse the variability of the CST reconstruction either based on non-gated or on cardiac-gated data sets. Therefore, for each volunteer and for each group (non-gated, cardiac-gated), the intersection and union of the reconstructed CST objects was calculated to define the corresponding JD. To further quantify local variability, the CST was also subdivided into three parts, the brain stem (BS), the posterior limb of the internal capsule (PLIC) and the area above the corpus callosum (subcortex, SC). The JD was calculated accordingly.

### Statistical analysis

Statistical analysis, except for TBSS analysis, was performed in SPSS Statistics 24.0 (IBM, Armonk, USA) using independent and paired t-tests. The significance level was set to p < 0.05.

### Data availability

The data sets generated during and/or analysed during the current study are available from the corresponding author on reasonable request.

## Results

### Analysis of motion artefacts and signal variability in DWI data

**Figure 1:**
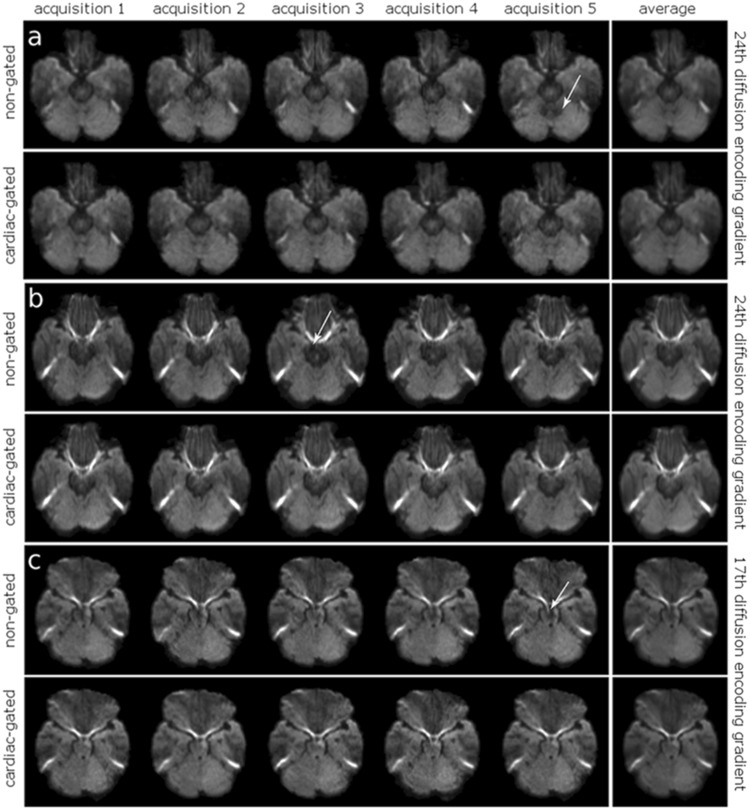
Visual Inspection of pulsatile motion artefacts in single volunteers. Visual inspection of pulsatile motion artefacts (24^th^ diffusion encoding gradient, smallest deviation from the z-axis) shows exemplary obvious artefacts (white arrow) in the superior cerebellum and the mesencephalon in non-gated data of two volunteers (**a**: left upper row, **b**: left upper row) in contrast to cardiac-gated data (**a**: left bottom row, **b**: left bottom row), almost disappeared after image averaging (**a**: right column, **b**: right column). In case of the 17^th^ diffusion encoding gradient (largest deviation from the z-axis) obvious artefacts are seen in the mesencephalon in non-gated data (**c**: left upper row), not visible in the cardiac-gated data (**c**: left bottom row) and after averaging (**c**: right column).

In case of the non-gated DWI data, in the image sets with smallest deviation of the diffusion encoding gradient from the z-axis (volume 24), obvious artefacts were found within the superior cerebellum and the mesencephalon in thirteen volunteers (Fig. 1a,b). Artefacts thereby occurred randomly in a subset of the five repetitive non-gated DWI data sets, with an artefact occurrence ratio of 11.82% calculated as number of data sets including obvious alterations divided by the total amount of data sets (22 × 5). After image averaging, no obvious artefacts remained. For the image sets of the non-gated DWI data with largest deviation of the diffusion encoding gradient from the z-axis (volume 17), obvious artefacts were present in five volunteers within the mesencephalon (Fig. 1c). The artefact occurrence ratio was 4.55%. After image averaging the artefacts disappeared. In case of the cardiac-gated DWI data no obvious artefacts were found neither in the single data sets nor the averaged images.

**Figure 2:**
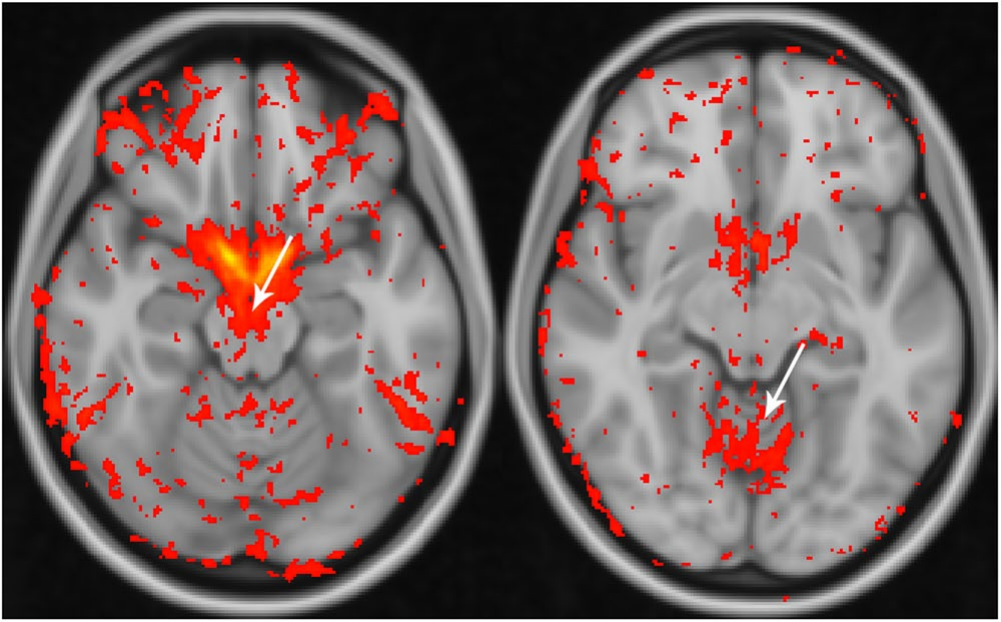
Standard deviation differences across non-gated and cardiac-gated data. Analysis of the standard deviation across non-gated and cardiac-gated data. Note that a higher standard deviation within the mesencephalon (left) and superior cerebellum (right) is seen in the non-gated data also on group level (red to yellow colour scheme with small differences encoded in red and large differences encoded in yellow).

Analysis of the SD maps revealed obviously higher SD values in the non-gated image sets within the mesencephalon and the superior cerebellum (Fig. 2).

### Diffusion tensor properties and parameters along the CST

**Figure 3:**
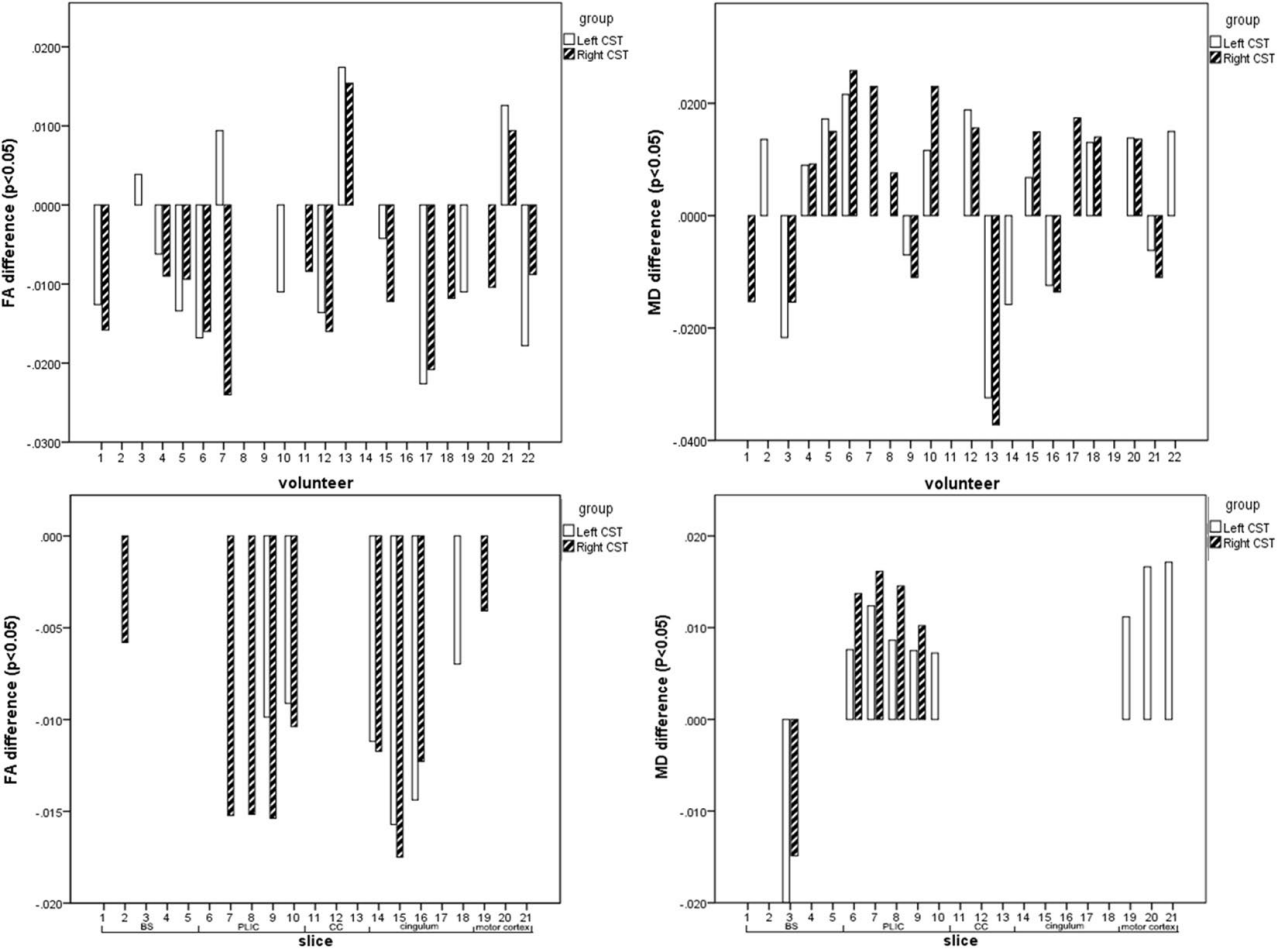
Analysis of FA and MD within the masked CST. Top: General analysis of FA and MD within the masked CST for each volunteer showing significant FA differences (left) and MD differences (right) between the non-gated and cardiac-gated imaging data. Bottom: Analysis of FA- and MD-differences within the CST between the non-gated and cardiac-gated data sets within defined sections: brainstem (slices 1–5), posterior limb of internal capsule (slices 6–10), corpus callosum (slices 11–13), cingulum (slices 14–18), motor cortex (slices 19–21).

At single subject level, more volunteers showed smaller overall FA values and bigger overall MD values within the cardiac-gated DWI data sets compared to non-gated DWI data sets. For every volunteer the difference in FA values between non-gated and cardiac-gated data ranged from -0.004 to -0.024, for MD it ranged from 0.007 × 10^−3^ to 0.026 × 10^−3^ (Fig. 3, top).

On group level, within the cardiac-gated DWI data sets the mean FA value was 0.49 ± 0.02 for the left CST and 0.45 ± 0.02 for the right CST. Based on the non-gated DWI data sets mean FA value was 0.47 ± 0.02 for the left CST and 0.46 ± 0.02 for the right CST. In case of the right CST, a significant difference (p < 0.01) in overall FA values between non-gated and cardiac-gated images was found, whereas for the left CST no significant difference (p = 0.08) was found. In case of mean diffusivity in the cardiac-gated data sets a mean MD value of 0.82 × 10^−3^ ± 0.04 × 10^−3^ for the left CST and 0.85 × 10^−3^ ± 0.04 × 10^−3^ for the right CST was found. In the non-gated data sets the mean MD value was 0.82 × 10^−3^ ± 0.03 × 10^−3^ in the left CST and 0.85 × 10^−3^ ± 0.04 × 10^−3^ in the right CST. No significant difference between both groups was seen (p = 0.42 for left CST and p = 0.30 for right CST).

The slice based analysis demonstrated significantly smaller FA values (p < 0.05 for all slices) at the level of BS and PLIC (differences ranging from -0.008 to -0.016) and significantly bigger MD values (p < 0.05 for all slices) at the level of PLIC (ranging from 0.007 × 10³ to 0.018 × 10^−3^) (Fig. 3, bottom).

### TBSS analysis

**Figure 4:**
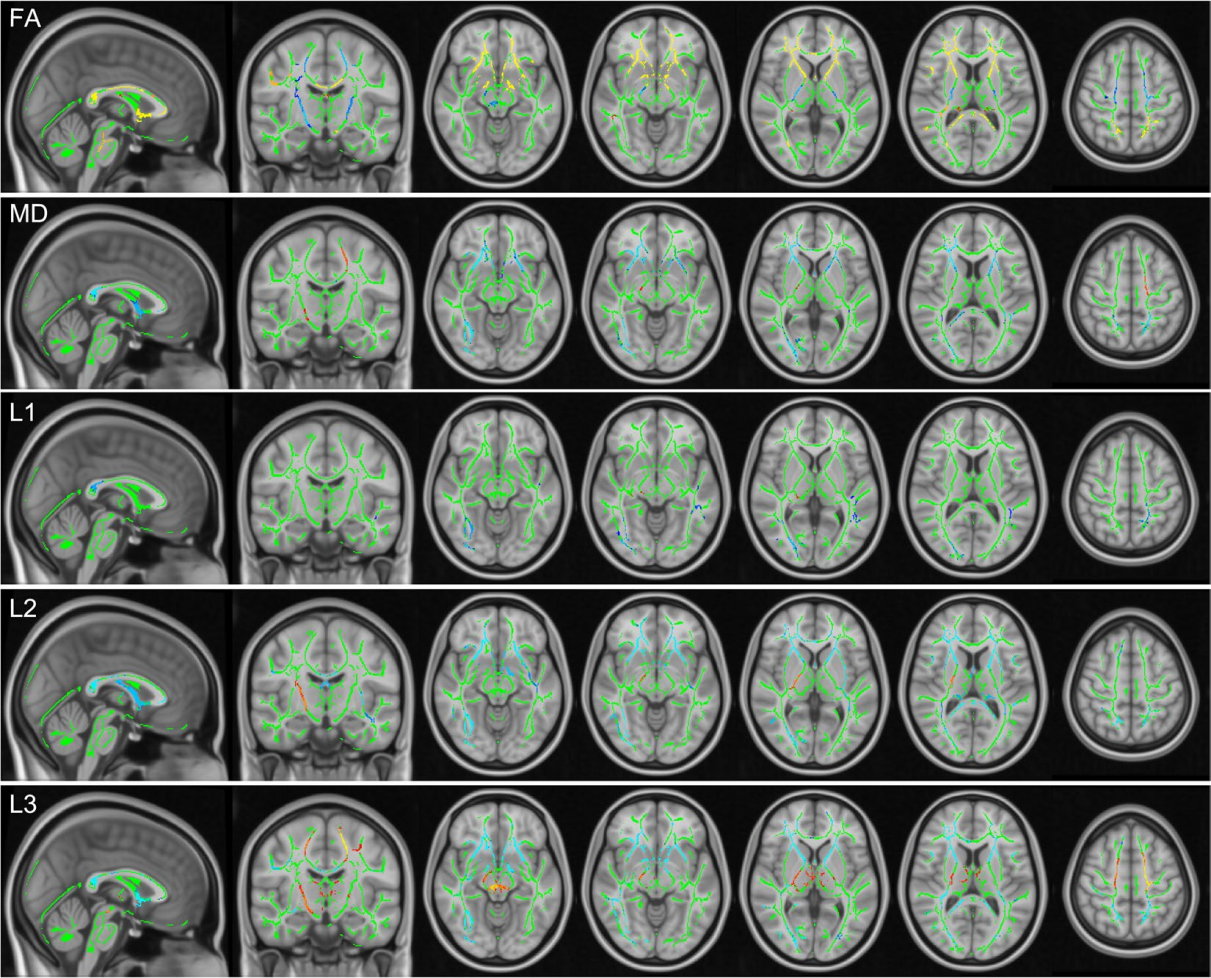
TBSS analysis of scalar diffusion tensor parameters. Results of TBSS analysis of FA, MD and all three eigenvalues L1, L2, L3 between non-gated and cardiac-gated image data with the fiber tract skeleton visualized in green, significantly higher (blue) and smaller (yellow to red) parameter values within the non-gated group.

Using TBSS, alterations according to FA, MD and the three eigenvalues L1, L2, L3 were analysed within the whole brain (Fig. 4). Table 1 summarizes significant changes according to FA, MD, L1, L2 and L3. Across the whole brain, cardiac-gated data showed significant (p < 0.05) changes within the CST, with smaller FA values in BS, PLIC and SC. Significant alterations were accordingly seen within the BS and PLIC with larger MD values (p < 0.05). No significant difference between non-gated and cardiac-gated data was seen for L1. In case of L2, significantly (p < 0.05) larger values were seen in the BS and PLIC, whereas significantly (p < 0.05) larger values of L3 were seen across all three regions (BS, PLIC, SC). Comparing both hemispheres, larger clusters of significant differences were seen within the right hemisphere.

**Table 1:** TBSS analysis of scalar diffusion tensor parameters.

Location	FA	MD	L1	L2	L3
BS	−(R)	+(R)	/	+(R)	+(R)
PLIC	−	+(R)	/	+(R)	+
SC	−	/	/	/	+
Genu CC	+	−	/	−	−
Splenium CC	+	−	−	−	−
ALIC	+	−	/	−	−

TBSS based significant alterations of FA, MD, L1, L2 and L3 of cardiac-gated data in relation to non-gated data (R: only within the right hemisphere).

Besides the CST, also the corpus callosum (CC) was significantly affected by pulsatile motion (p < 0.05). Cardiac-gated data revealed significantly larger FA values within the genu and splenium of the CC, whereas significantly smaller values were found for MD, L2, and L3. The anterior limb of the internal capsule (ALIC) was affected in the same way.

### The principal eigenvector

**Figure 5:**
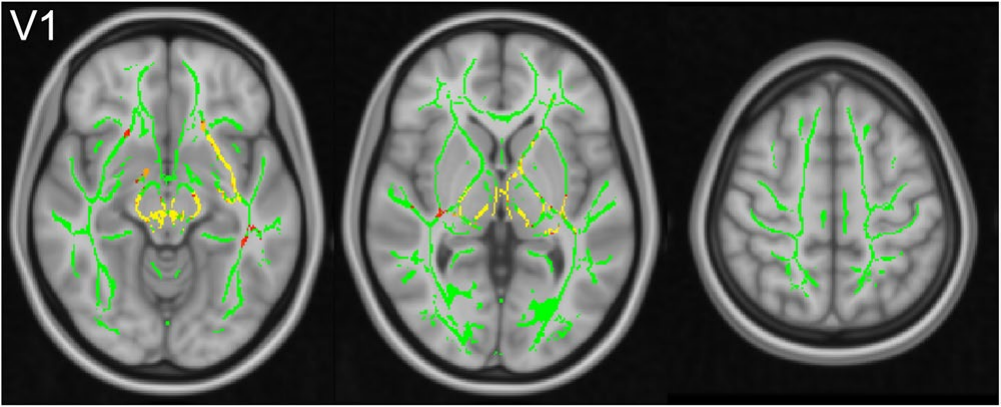
TBSS analysis of the principal eigenvector. TBSS analysis shows significantly larger deviation of the principal eigenvector from the z-axis for cardiac-gated data (fiber tract skeleton visualized in green, significantly larger angles visualized in yellow to red).

**Figure 6:**
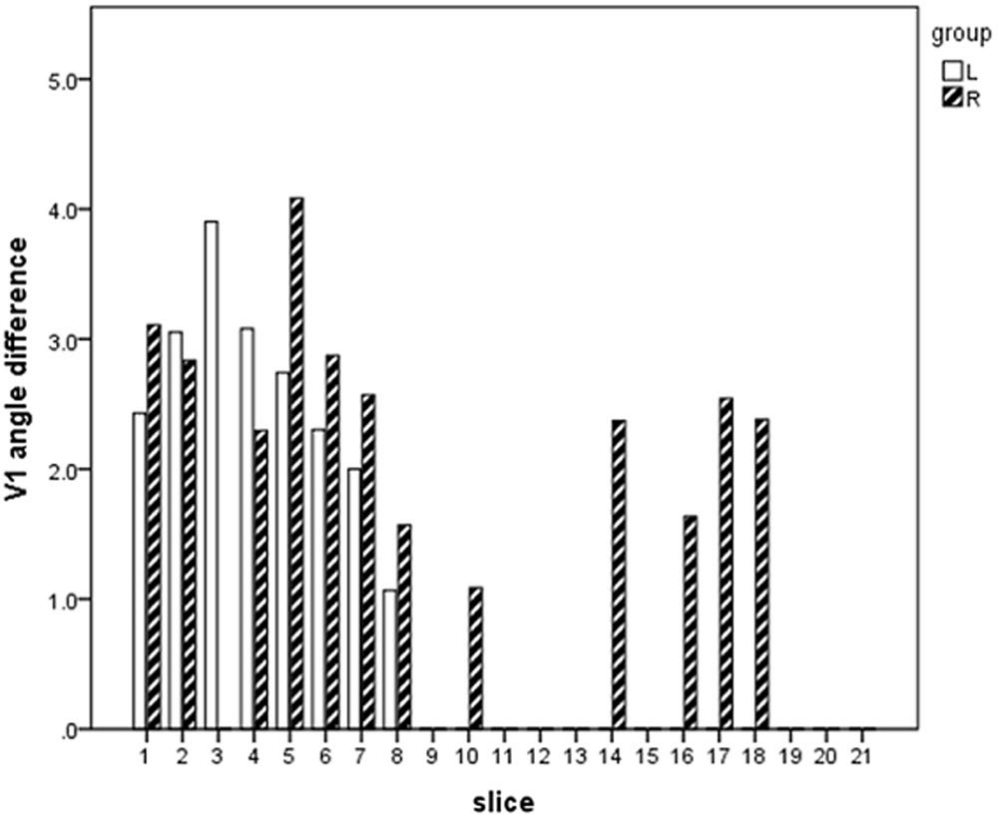
Deviation of principal eigenvectors across the CST. Significant angle differences across slices between non-gated and cardiac-gated data within defined sections: brainstem (slices 1–5), posterior limb of internal capsule (slices 6–10), corpus callosum (slices 11–13), cingulum (slices 14–18), motor cortex (slices 19–21).

TBSS analysis of the deviation of the principal eigenvector V1 from the z-axis showed a significant increase (p < 0.05) within the bilateral BS and PLIC in case of the cardiac-gated data (Fig. 5). Using the slice-based approach to estimate the effect on the principal eigenvector of non-gated and cardiac-gated data on different levels of the CST, a significant deviation (ranging from 1° to 4°) was found within the BS and PLIC in both hemispheres. At the level of the CC, the cingulum and motor cortex, no significant differences were found within the left hemisphere at the level of CC, cingulum and motor cortex. Within the right hemisphere a significant change was seen at the level of the cingulum (Fig. 6).

### Fiber Tractography of the CST

After tractography, the CST volume in the hemisphere was 13.72 ± 2.38 cm^3^ (non-gated) and 13.84 ± 2.32 cm^3^ (cardiac-gated), respectively (p = 0.55). The tract volumes ranged from 7.37 cm^3^ to 18.83 cm^3^ in case of non-gated data and from 8.50 cm^3^ to 19.44 cm^3^ in case of cardiac-gated data. The CST volume in the right hemisphere was 12.69 ± 2.20 cm^3^ for non-gated image data and 12.35 ± 2.26 cm^3^ for cardiac-gated data (p = 0.37). Tract volumes ranged from 7.55 cm^3^ to 16.98 cm^3^ (non-gated) and 6.37 cm^3^ to 17.44 cm^3^ (cardiac-gated), respectively.

### Tract variability

For the left CST the tract dissimilarity was 0.52 ± 0.11 (non-gated) and 0.54 ± 0.07 (cardiac-gated), respectively, showing no significant difference (p = 0.41). The right CST revealed tract dissimilarities of 0.53 ± 0.12 (non-gated) and 0.57 ± 0.10 (cardiac-gated), respectively (p = 0.06). For the subdivisions of the left CST dissimilarity measures of 0.53 ± 0.14 (non-gated) vs. 0.54 ± 0.08 (cardiac-gated) (p = 0.53) for the brain stem, 0.47 ± 0.08 (non-gated) vs. 0.48 ± 0.08 (cardiac-gated) (p = 0.44) for the PLIC and 0.57 ± 0.16 (non-gated) vs. 0.56 ± 0.09 (cardiac-gated) (p = 0.77) were seen. In case of the right CST, tract dissimilarities resulted in values of 0.54 ± 0.13 (non-gated) vs. 0.56 ± 0.10 (cardiac-gated) (p = 0.53) for the brain stem, 0.48 ± 0.11 (non-gated) vs. 0.51 ± 0.09 (cardiac-gated) (p = 0.19) for the PLIC and 0.53 ± 0.13 (non-gated) vs. 0.60 ± 0.12 (cardiac-gated) (p = 0.02). For further details see Table 2.

**Table 2:** Tracts volume and Jaccard distances along the CST.

	hemisphere	group	tract volume (mean ± SD)	p	JD (mean ± SD)	p
CST	L	NG	13.72 ± 2.38	0.55	0.52 ± 0.11	0.41
		G	13.84 ± 2.32		0.54 ± 0.07	
	R	NG	12.69 ± 2.20	0.37	0.53 ± 0.12	0.06
		G	12.35 ± 2.26		0.57 ± 0.10	
BS	L	NG	2.51 ± 0.49	0.48	0.53 ± 0.14	0.53
		G	2.45 ± 0.60		0.54 ± 0.08	
	R	NG	2.45 ± 0.46	0.13	0.54 ± 0.13	0.53
		G	2.32 ± 0.48		0.56 ± 0.10	
PLIC	L	NG	3.23 ± 0.70	0.06	0.47 ± 0.08	0.44
		G	3.12 ± 0.61		0.48 ± 0.08	
	R	NG	3.01 ± 0.59	0.15	0.48 ± 0.11	0.19
		G	2.84 ± 0.46		0.51 ± 0.09	
SC	L	NG	7.96 ± 1.67	0.18	0.57 ± 0.16	0.77
		G	8.28 ± 1.74		0.56 ± 0.09	
	R	NG	7.61 ± 1.59	0.26	0.53 ± 0.13	0.02
		G	7.31 ± 1.61		0.60 ± 0.12	

Tract volume and Jaccard distances (JD) along the CST and its subdivisions (BS, PLIC and SC) based on non-gated (NG) and cardiac-gated (G) image sets.

## Discussion

DWI, DTI and DTI based fiber tractography are becoming increasingly important for various applications. For neurosurgical applications, a precise and accurate visualization of major white matter tracts is highly relevant, so that acquisition of high-quality and reliable DWI data deserves great attention. Pulsatile motion, greater than Brownian motion typically measured during DWI data acquisition, has in part been reported to produce motion artefacts and to effect diffusion tensor parameters^7,13,17^. Other studies did not detect obvious motion artefacts and effects of pulsatile motion at all^14^. As there is still no consensus on the effects of pulsatile motion, especially in the corticospinal tract, which is an important structure in neurosurgical applications, we furthermore aimed to analyse the effects of pulsatile motion on different levels of processing (DWI data, tensor parameters, fiber tractography).

Initially, obvious image artefacts within the co-registered non-gated and cardiac-gated DWI images of each volunteer were analysed within two volumes with diffusion encoding gradient directions with smallest and largest deviations from the z-axis. Obvious artefacts thereby occurred only for non-gated data in mesencephalon and cerebellum. In the data with diffusion encoding gradient direction most similar to the z-axis, more artefacts arise, whereas artefacts were randomly scattered over single data sets. The occurrence ratio was about 4.55% to 11.81%, consistent with previous reports (6% to 20%)^10,12^. As in cardiac-gated images no obvious artefacts were detected, cardiac-gating could help to reduce those artefacts leading to signal attenuation.

Looking at the signal variability using the SD maps, larger signal variation was also seen in the mesencephalon and cerebellum for non-gated images. Previous studies reported obvious motion artefacts in brain stem, cerebellum, thalamus, and the corpus callosum^9,27^. Significant artefacts were seen in image sets with diffusion encoding gradient direction along the z-axis^7,12,13,27^ but more pronounced with larger signal attenuation than in our study data. Effects of pulsatile motion thereby might not only arise for diffusion encoding gradient directions almost parallel to the z-axis but also for other diffusion encoding gradient directions. Until now, only a single report analysed DWI signal artefacts along 16 directions, in case of a 1-month old infant. The signal artefacts occurred in brain stem and cerebellum and were not obvious^39^. As in our study the closest diffusion encoding gradient direction along the z-axis showed deviations of 18.77° ± 5.42°, the reported effect might be reduced.

As there are obvious effects on the signal intensities in DWI data sets, the next step was to analyse the effects on diffusion tensor parameters, relevant for neuroscientific analyses and fiber tractography. There is only a small number of articles investigating less volunteers reporting changes of FA and MD within the CC, the thalamus, or the midbrain^7,15,40^, but these are not focused on a particular white matter tract or even explored the principal eigenvectors’ change. In this study we focused on the CST. The general analysis of diffusion tensor parameters as well as the TBSS analysis demonstrated that pulsatile motion caused an over-estimation in FA and under-estimation in MD. Cardiac-gating decreased FA and increased MD. Specifically, an over-estimation of FA was seen in the BS, PLIC and SC, an under-estimation of MD within the BS and PLIC. Further analysis revealed that the effect on FA and MD was heavily influenced by the second and third eigenvalue, rather than by the first eigenvalue. Even though there was larger pulsatile motion, the effects on the first eigenvalue remained non-significant.

We also observed a difference between both hemispheres with significant effects of cardiac-gating on the FA value along the right CST. The FA value of the left CST was larger than of the right CST which is consistent with previous report on CST asymmetry^41^. Maybe the higher tract anisotropy within the left hemisphere leads to less effects of pulsatile motion. In other locations, such as the CC and ALIC, the result was the opposite. Even though tract anisotropy is similarly high in CC, ALIC and PLIC, tract direction also seems to play an important role with tract direction of anterior-posterior (ALIC), superior-inferior (PLIC) and left-right (CC). As the pulsatile motion is very complex the direction of pulsatile motion can be quite different in distinct regions of the brain and might thereby cause different effects on regions of different fiber tract directions.

Significant changes (increase) of the deviation of the principal eigenvector V1 from the z-axis were seen for the cardiac-gated data in the BS and PLIC. This is in accordance with previous reports observing a change in the principal eigenvector’s direction within the cerebellum of 27°^39^, the CC and transverse fibers in the BS of less than 5°^42^. Although pulsatile motion did not affect the length of the principal eigenvector, its direction seems to be affected.

**Figure 7:**
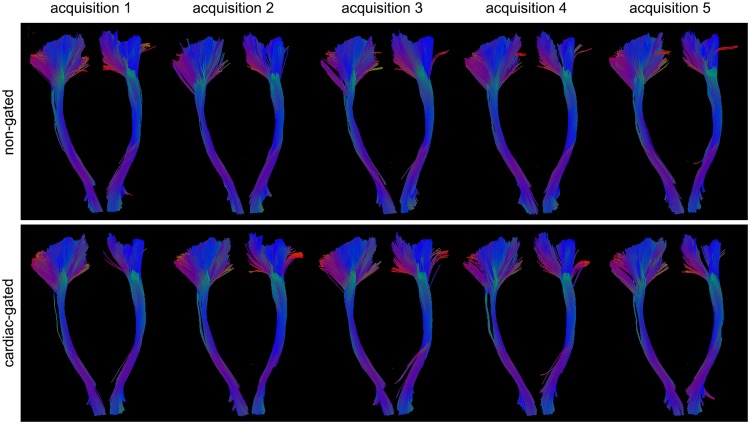
Illustrative case of fiber tractography of the CST. Fiber tractography of the left and right CST of one healthy volunteer for non-gated and cardiac-gated data across all five acquisitions.

After analysis of DWI signal intensities, diffusion tensor properties and parameters, finally the effects on fiber tractography are considered. As anatomical correctness of fiber tractography can be evaluated by intraoperative subcortical stimulation or post mortem data, not in healthy volunteers, we focussed on the reconstructed tract volume and only visually inspected its anatomical plausibility during the fiber tracking procedure. Visual inspection of fiber tractography results of the CST thereby only revealed subtle differences (Fig. 7). Regarding the fiber tract volumes, also no significant difference between tractography based on non-gated or cardiac-gated images was seen, although pulsatile motion caused image artefacts, changed diffusion tensor parameters and also altered the direction of the principal eigenvector. In our study we considered a DWI acquisition scheme with 30 diffusion encoding gradient directions^43^, rather than the minimum prerequisite of six diffusion encoding gradient directions, typically used in older studies. Thereby higher angular resolution (i.e. increased number of gradient directions) can lead to decreased tractography variation and improvement of localization accuracy^41,44^. Previous articles on the effect of pulsatile motion on diffusion tensor parameters investigated only six to 15 diffusion encoding gradient directions. In particular the report focusing on the effect on tractography, using a probabilistic approach, investigated only six diffusion encoding gradient directions, making it possibly more sensitive to effects of pulsation artefacts^15,40^. Even though spatial resolution affects spatial accuracy it has been proved that angular resolution is more effective than spatial resolution and can thereby decrease the variation along white matter tracts^41^.

Considering the tract variation of the CST investigating the Jaccard distance, no obvious difference was observed between tracts based on non-gated or cardiac-gated DWI data sets for the whole CST. This is in accordance with our previous results on pulsatile motions effects on the FA values along the CST (see above).

There is still no consensus on the real necessity of cardiac-gated DWI data acquisition for DWI, DTI and fiber tractography. While some reports show no effect of pulsation at all, some alternative reports consider cardiac-gating as an option to overcome artefacts and signal attenuation due to pulsation. Additionally there are other authors reporting on the effects of pulsatile motion, but applied other techniques to overcome those effects without cardiac-gating, e.g. multi-scanning, image averaging, different acquisition techniques (Turboprop DTI), zero-padding reconstruction or full Fourier acquisition approaches (both insensitive to motion) or filtering algorithms to remove motion associated noise^10,27,45–49^. Among these approaches, image averaging seems appropriate to overcome these effects in the clinical routine. In accordance with this, besides the high angular resolution, we also observed no differences in signal intensities in the DWI data after image averaging between the cardiac-gated and non-gated group.

Although averaging (even though done after tensor estimation) dilutes the random occurrence of motion artefacts, it did not eliminate the differences between both groups according to FA, MD and the direction of the principal eigenvector V1. Thus, averaging cannot substitute cardiac-gated data acquisition completely, even though averaging leads to more calculable acquisition times than cardiac-gated acquisition.

There are four known sources of pulsatile motion including arterial pulse, venous expansion, CSF flow and capillary expansion. Phase-velocity measurements have shown that highest velocities are present in the inferior and medial areas of the brain and decreasing towards the periphery^9^. Motion caused by microvascular or capillary expansion typically occurs in parenchyma, also called intra-voxel incoherent motion (IVIM)^50–53^. This type of motion has been described early and represents perfusion parameters to some degree. It can be observed only when the b-value is lower, e.g. below 1000 s/mm². In this study, we obtained b-values of 1000 s/mm². Thus, IVIM did not contribute to the motion effect. Arterial pulse, venous expansion and CSF flow occurs in cranial base and medial part of brain. The motion artefacts we observed belong to these regions.

## Conclusion

Reconstruction and highly accurate visualization of the CST is of great importance in neurosurgical applications. We analysed the effect of pulsatile motion on the CST ranging from signal intensities, diffusion tensor parameters to fiber tractography. Although obvious signal changes and effects on diffusion tensor parameters and the principal eigenvector were seen, pulsatile motion did not significantly influence the fiber tract reconstruction of the CST regarding tract volume nor obviously affected spatial location with clinical standard algorithms. State-of-the-art image acquisition with high angular resolution and averaging already seems to overcome the effects of pulsation on the reconstruction of the CST.
